# Knowledge and perceptions about the health impact of climate change among health sciences students in Ethiopia: a cross-sectional study

**DOI:** 10.1186/1471-2458-14-587

**Published:** 2014-06-11

**Authors:** Andualem S Nigatu, Benedict O Asamoah, Helmut Kloos

**Affiliations:** 1 International Master Programme in Public Health, Faculty of Medicine, Lund University, CRC, Jan Waldenströms gata 35, 205 02 Malmö, Sweden; 2 Master Program in Science for Sustainable Development, Department of Water and Environmental Studies, Linköping University, Linköping, Sweden; 3 Department of Epidemiology and Biostatistics, University of California, San Francisco, USA

**Keywords:** Climate change, Ethiopia, Climate related human health impacts, Knowledge and perception

## Abstract

**Background:**

Climate change affects human health in various ways. Health planners and policy makers are increasingly addressing potential health impacts of climate change. Ethiopia is vulnerable to these impacts. Assessing students’ knowledge, understanding and perception about the health impact of climate change may promote educational endeavors to increase awareness of health impacts linked to climate change and to facilitate interventions.

**Methods:**

A cross-sectional study using a questionnaire was carried out among the health science students at Haramaya University. Quantitative methods were used to analyze the results.

**Result:**

Over three quarters of the students were aware of health consequences of climate change, with slightly higher rates in females than males and a range from 60.7% (pharmacy students) to 100% (environmental health and post-graduate public health students). Electronic mass media was reportedly the major source of information but almost all (87.7%) students stated that their knowledge was insufficient to fully understand the public health impacts of climate change. Students who knew about climate change were more likely to perceive it as a serious health threat than those who were unaware of these impacts [OR: 17.8, 95% CI: 8.8-32.1] and also considered their departments to be concerned about climate change (OR: 7.3, 95% CI: 2.8-18.8), a perception that was also significantly more common among students who obtained their information from the electronic mass media and schools (p < 0.05). Using electronic mass media was also significantly associated with knowledge about the health impacts of climate change.

**Conclusion:**

Health sciences students at Haramaya University may benefit from a more comprehensive curriculum on climate change and its impacts on health.

## Background

There is a growing concern among health officials and policy makers about climate change and its potential impact on the environment and human health [1]. The potential impact of climate change on population health is enormous [1] and is expected to worsen unless efforts are made to curb the emission of greenhouse gases into the atmosphere [2]. Climate change affects human health and wellbeing in several ways, notably by increasing the distribution and transmission of several vector-borne infectious diseases, including malaria, dengue and leishmaniasis, and diseases related to heat stress, by increasing losses of human lives, livestock and property through severe floods and droughts, and by reducing the availability of water for domestic uses, sanitation levels, as well as losses in food production, biodiversity and ecosystem functions [1]. Climate-linked shortages of domestic water supplies can negatively affect personal hygiene and environmental sanitation, increasing diarrheal, skin and eye diseases [3]. According to the World Health Organization [4], the impact of recent climate change has already caused the loss of 150,000 human lives and about 5 million DALYs (Disability Adjusted Life Years) throughout the world. Accelerating demographic, social, economic, environmental and ecological changes as a result of increasing globalization and the inability of many developing countries and their populations to make the necessary adaption changes to mitigate the effect of climate change [5,6] renders Africa particularly vulnerable to the effects of climate change. Climate change thus presents a new face and a challenging scenario in the battle to improve global health overall and health in Africa in particular.

The magnitude and effects of climate change have been estimated with increasing accuracy and reported from different regions of the world. Heat waves are now a common event in Europe and caused the death of thousands of elderly people in 2003 [7]. The effects of climate change are predicted to increase in Europe in the coming decades [8]. Asia is increasingly experiencing extensive flooding resulting in high human mortality, injuries, water-borne diseases and mental health problems [9-11]. According to the Asia Development Bank [12], 30 million Indians were affected by flooding in 2007. The global burden of diseases caused by climate change is likely to increase in the future. It is estimated that the health of millions of people throughout the world will be affected due to increases in malnutrition resulting from food shortage; diseases and injuries associated with heat waves, floods, storms, fires and droughts; increases in gastrointestinal diseases and cardio-respiratory disease, and altered distribution and seasonal transmission of vector-borne disease [2].

Africa is the continent most vulnerable to the impacts of climate change, due to increases in both droughts and floods, the epidemic potential of many infectious diseases and low capacity of nations and communities to adapt to climate change [6,13]. Regular, predictable rainfall is particularly critical in Africa because many countries are highly dependent on rain-fed agriculture. According to the climate change scenario described by the United Nations Framework Convention on Climate Change in 2007, agricultural productivity will decline and increase food insecurity. The production of subsistence crops in Ethiopia, Eritrea, Ghana, Zambia and Sudan has already decreased in the recent years, exacerbating hunger and malnutrition-linked diseases. Moreover, Africa is facing increasing water scarcity and associated increases in some water-related diseases [6,13].

Ethiopia is one of the countries vulnerable to climate change [14] and losses of human lives from flooding, domestic water supply shortages, malnutrition and the altitudinal extension of malaria transmission are increasingly being reported. Flooding is one of the climate related hazard that affects human health in several ways such as death, injuries, water-borne diseases, malnutrition, and mental ill health [10,14]. According to the 2007 report of the National Adaptation Program of Action [15], major losses of human lives and property were reported from different parts of Ethiopia in 1988, 1993, 1994, 1995, 1996 and 2006. For instance, in 2006, flooding took 256 human lives in Dire Dawa Town and 364 lives in South Omo Zone. More than 10000, 6000 and 16000 inhabitants of Dire Dawa, South Omo, and West Shewa zones, respectively were become homeless due to river flooding in 2006. In addition, millions of dollars worth of property were damaged in Dire Dawa, and about 10,000 livestock of the Afar ethnic groups perished in the floods [15].

Climate change is causing domestic water supply shortages, which in turn can affect personal hygiene and environmental sanitation, increasing exposure to diarrheal diseases [3]. Cases of reported water-borne diseases, such as unspecific dysentery and diarrheal cases, increased in Ethiopia from 19,980 in 2008 to 116,571 in 2011 [16,17]. Malnutrition is one of the climate related impacts that affects mainly the growth of children, and the distribution of malnutrition among children in Ethiopia has been associated with the occurrence of drought. Based on climate change projections, annual mean temperatures are projected to increase over the coming decades in Ethiopia [14,17]. These changes could contribute to malnutrition among under- five children. Increasing temperatures are also facilitating the spread of vector- borne diseases, particularly malaria, from lower altitudes to the Ethiopian highlands as a result of increasing temperatures [14,15,18].

Despite many publications on the preparedness and assessment of knowledge and perception of climate change and health impacts among local health officers [19-21], previous studies failed to examine the knowledge and perception of health sciences students on climate change related health impacts. The objective of this study is to assess the knowledge and perceptions of health sciences students of Haramaya University in Ethiopia regarding the impact of climate change on human health.

## Methods

### Study area and population

This study was carried out in the College of Health Science of Haramaya University, Harar campus. Haramaya University, located in eastern Ethiopia, was established in 1955 and currently has 10 colleges and 49 study programmes. The College of Health Sciences was established in 1995/1996 and offers 10 programmes, namely Medical Laboratory Technology, Environmental Health, Health Officer, Nursing, Midwifery, Psychiatry Nursing, Pharmacy, Medicine, and Public Health. The study subjects were recruited all these programmes (Table 1).

**Table 1:** Demographic characteristics and departmental affiliation of 306 health sciences students of Haramaya University, 2011

**Demographic characteristics**	**Number**	**Percentage**
Sex:		
Male	189	6l.8
Female	117	38.2
Total	306	100
Age:		
18-24 yrs	182	59.5
25-31 yrs	116	37.9
≥32 yrs	8	2.6
Total	306	100
Programmes/departments of students:		
Medical laboratory	8	2.6
Nursing	55	18.0
Health officer	105	34.3
Environmental health	6	2.0
Psychiatric nursing	13	4.2
Medicine	54	17.6
Midwifery	28	9.2
Pharmacy	28	9.2
Public health (post-graduates)	9	2.9
Total	306	100

### Variable definition and measurement

Knowledge and perception are the dependent variables selected to evaluate the level of student awareness of the relationship between climate change and health and the commitment to address this issue. Knowledge was measured by using the scoring system by Mpazi and Mnyika [22] and Abdeyazdan and Sedeghi [23]. For each correct answer a score of one point was given and a score of zero point for incorrect answers. The mean of students’ scores was taken as cut-off point in identifying students with good and those with poor knowledge of climate change. Students scoring above the mean (correctly answering a minimum of 7 out of the 14 questions) were regarded as knowledgeable while those who scored below the mean were classified as having poor knowledge about climate change. Knowledge questions included were about awareness of climate change, students’ knowledge of the effect of climate change at their place of residence, health impacts of climate change, and possible effects of climate change.

Questions on perception were formulated to evaluate student responses to questions about the impact of climate change on human health. Students were asked how they perceived the threat of climate change impacts, their role as health professionals in the prevention of public health impacts of climate change, and whether they considered their respective departments to be concerned about the prevention of climate change.

### Sampling procedure

All 327 students enrolled in nine health-related departments and programmes of Haramaya University were chosen for the study (see Table 2). This sample size satisfies the requirement for the formula for estimating a single population proportion with the assumption that the maximum proportion of students aware of climate change health impact is (p = 0.5) with 95% confidence interval and 5% marginal error.

**Table 2:** Level of knowledge about climate change health impacts among 306 health sciences students of Haramaya University, by department or program, 2011

**Variables**	**Clinical nursing (%)**	**Medical laboratory technology (%)**	**Envir. health (%)**	**General nursing, (psychiatric nursing and midwifery) (%)**	**Medical (%)**	**Pharmacy (%)**	**Public health (Post-grad.) (%)**	**Health officer (%)**	**Age(<25 yrs) (%)**	**Age( ≥ 25 yrs) (%)**	**Males (%)**	**Females (%)**
Good knowledge	40(72.7)	6(75.0)	6(100)	32(78.0)	38(70.4)	17(60.7)	9(100)	89(84.8)	100(80.7)	137(75.3)	145(76.7)	92(78.6)
Poor knowledge	15(27.3)	2(25.0)	0	9(22.0)	16(26.6)	11(39.3)	0	16(15.2)	45(19.3)	45(24.7)	44(23.3)	25(21.4.4)

A proportionally stratified sampling procedure was used to select the study subjects. This kind of sampling procedure can guarantee the representation of each of the eight groups and also their subgroups (the number of 1^st^, 2^nd^, 3^rd^ and 4^th^ year’s student of a given programme year) to ensure that the subjects were sampled proportionally. The study includes both BSc and MSc health science students. The number of study participants from each programme was determined by dividing the number of students within the programme by the total number of students in the College of Health Sciences (2190) and multiplying by the sample size (327). Then the number of participating students in each study year was determined by dividing these numbers by the total number of students in the College of Health Sciences in each study year by the total number of students attending the programme multiplied by the required number of students from each respective programme.

In order to select the individual students who participated in the study, the students’ identity (ID) numbers were entered into a computer software programme (Random Number Generator Software) which generated at random the required number of students from each programme and within a study year. The students whose ID numbers were selected by the computer programme were invited to participate in the study.

### Data collection

A self- administered questionnaire consisting of 26 closed-ended questions was first developed in English and then pre-tested among 33 (about 10% of the study population) non-selected students for assessing content validity, appropriateness, and comprehensibility of the questions. The edited questionnaire was administered by the principal investigator to the 306 participating students in a lecture hall of Haramaya University from January 28 to February 25, 2011. The questionnaire consisted of two parts. The first part contained questions about the demographic characteristics of the study participants and the second part focused on climate change and its impacts on health.

### Data analysis

Data collected during the survey were checked in the field before they were entered and analyzed using SPSS, Version 19. After descriptive analysis of demographic and social characteristics of the students, a chi-square test was used to examine whether or not an association existed between students’ awareness of the impact of climate change and students’ concerns about preventing the public health consequences of climate change. A p-value of <0.05 was considered to indicate a statistically significant difference. Bivariate logistic regression was subsequently performed to examine the relationship between variables.

### Ethical consideration

The study obtained ethical clearance from the Institutional Review Board of Haramaya University. Prior to administering the questionnaire, the purpose of the study was briefly explained to and written informed consent obtained from voluntary study participants.

## Results

Out of the 327 students sampled for the study, 306 (93.6%) completed the questionnaire. Nine (2.7%) students refused to participate while 12 (3.7%) did not show up at the lecture hall to complete the questionnaire. The demographic characteristics of the study participants are described in Table 1. The mean age of the study participants was 24.1 years (±2.3SD). In this study the participation of males (61.8%) was higher than in females (38.2%). Most study participants were health officers (34.3%), followed by medical students (17.6%), and the smallest groups were environmental health students (2.0%).

**Figure 1:**
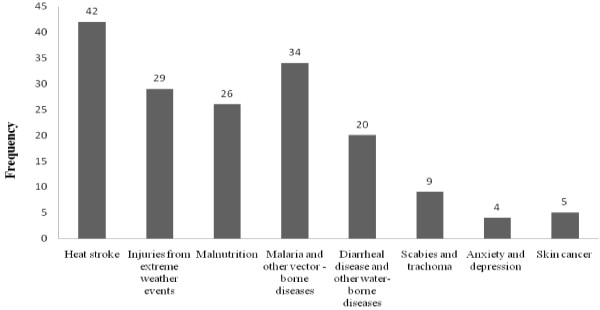
Frequency of responses by 306 Haramaya University health sciences students about different health effects of climate change, 2011.

**Figure 2:**
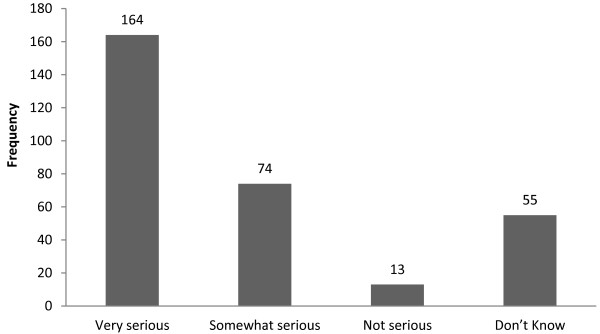
Perceptions of 306 Haramaya University health sciences students about the threats of climate change to human health, 2011.

Most of the respondents (237, 77.5%) were aware of climate change and its impacts, with similar rates for males (76.7%) and females (78.6%). Of the 237 students who were aware of climate change and its impacts, 107 (45.5%) identified flooding as the main extreme event, 60 (25.3%) named drought and 61 (25.7%) students mentioned increased temperatures. One hundred and thirty-two students (43.1%) had observed some kind of climate change impact in their home areas. Flooding, drought and increasing temperatures were mentioned as the most prominent climate change impacts by students living in Oromia and Amhara administrative regions.Most (63.1%) of the study participants identified electronic mass media (TV and radio) as the most common source of information about climate change while 47 (15.4%), 44 (14.4%), and 22 (7.1%) of them said that they had received information on climate change from schools, newspapers, and friends/neighbors, respectively. Newspapers are not published under school rather students indicated national newspapers as one of their sources of information on climate change. Only 169 students identify the specific health impact of climate change and heat stroke was commonly mentioned (24.8%), followed by malaria and other water-borne diseases (20.1%). Only 4 students (2.4%) mentioned anxiety and depression as possible climate change health risks (Figure 1).Slightly more than half (53.6%) of the 306 students believed that climate change could seriously impact human health, 74 (24.2%) somewhat seriously and 13 (4.2%) only lightly, while 55 (18.0%) did not know (Figure 2).

Two-hundred thirty-seven of the 306 students (77.5%) had a good understanding of climate change impacts and 69 (22.5%) had a poor understanding. Both the environmental health and post-graduate public health students scored 100% on the questions. A larger proportion of older students (75.3%) revealed good knowledge about climate change health impact than younger students (8.7%). The proportion of knowledgeable medical (70.4%) and pharmacy (60.7%) students was slightly lower than that of health officer (84.7%) students (Table 2).

The great majority of the students (269, 87.9%), claimed that they did not have the necessary knowledge to properly address climate change health impacts and 37 (12.1%) of them responded that they had the necessary knowledge to plan, implement and evaluate climate change related health impacts. Of the 269 students who claimed not to have the necessary knowledge, 90.3% cited inadequate training as the reason to address the health consequences of climate change. The remaining 26 (9.7%) students mentioned that they only had theoretical knowledge.

Table 3 shows the perception of the various health sciences students regarding whether their respective departments were concerned about preventing the public health consequences of climate change. Most health officer students (55.2%), environmental health students (83.3%) and public health students (88.9%) believed that their departments were concerned about preventing public health consequences of climate change while all medical and psychiatric nursing students responded that their departments were not concerned about climate change issues.

Chi-square tests revealed one statistically significant association, between students’ awareness of climate change and their perceived departmental role in preventing the public health consequences of climate change. Students who knew about climate change were more likely to perceive preventing climate change health impacts as one of their departments’ objectives than students who did not know about climate change (χ^2^_=_ 21.6, df 2, P < 0.05).

**Table 3:** Perceptions of 306 health science students in different programmes and departments of Haramaya University regarding the concern of their departments about prevention of climate change, 2011

		**Yes (%)**	**No (%)**	**Don’t know (%)**	**Total (%)**
Programme/department	Medical laboratory	1(12.5)	1(12.5)	6(75.0)	8(100)
	General nursing	7(12.7)	15(27.2)	33(60.1)	55(100)
	Health officer	58(55.2)	10(9.5)	37(35.3)	105(100)
	Environmental health	5(83.3)	0	1(16.7)	6(100)
	Psychiatric nursing	0	5(34.5)	8(65.5)	13(100)
	Medical	0	21(38.9)	33(61.1)	54(100)
	Midwifery	3(10.7)	11(39.3)	14(50.0)	28(100)
	Pharmacy	2(7.1)	12(42.9)	14(50.0)	28(100)
	Public health (post- graduates)	8(88.9)	0	1(11.1)	9(100)
	Total	84(27.5)	75(24.5)	147(48.0)	306(100)

Table 4 shows the results of bivariate logistic regression. The students who knew about climate change were 14 times more likely to know about its health impact than those students who didn’t know about climate change [OR: 14.1, 95% CI: 5.1-38.6]. Similarly, students who knew about climate change were more likely to perceive climate change as a serious threat to human health than those students who didn’t know about climate change [OR: 16.8, 95% CI: 8.8-32.1]. Students obtaining information on climate change from friends or neighbors were five times more likely to be aware about the impact of climate change than those who used newspapers as a source of information on climate change [OR: 5.1, 95% CI: 0.6-44.0]. Use of the mass media was significantly associated with both knowledge about the health impacts of climate change [OR: 2.9; 95% CI: 1.2-2.2] and the perception that the respective departments or programmes of students were concerned about the prevention of such impacts [OR: 4.3; 95% CI: 1.6-11.7], as was school as a source of information [OR: 9.7; 95% CI: 3.2-28.9] (Table 4).

**Table 4:** Crude odds ratio (OR) and confidence intervals (95% CI) obtained from bivariate logistic regression for 306 health sciences students of Haramaya University, 2011

**Demographic variables, sources of information, and climate change/health impacts**	**Knows about climate change**	**Climate change has an impact on human health**	**Climate change is a serious threat to human health**	**Is your department concerned about the prevention of health impacts of climate change?**
	OR(95% CI)	OR(95% CI)	OR(95% CI)	OR(95% CI)
Sex of the respondents				
Male	0.8(0.5–1.4)	1.7(0.9–3.3)	1.0(0.6–1.7)	1.6(0.9–2.6)
Female	1	1	1	1
Do you know about climate change?				
Yes		14.1(5.1–38.6)*	16.8(8.8–32.1)*	7.3(2.8–18.8)*
No	1	1	1	1
Study year:				
First	0.9(0.4–2.3)	2.9(1.0–8.3)	0.9(0.3–2.1)	1.2(0.5–2.9)
Second	1.2(0.5–3.0)	1.8(0.7–4.8)	1.5(0.6–4.0)	1.2(0.5–3.0)
Third	0.8(0.3–2.2)	1.8(0.6–5.2)	0.6(0.2–1.6)	1.3(0.5–3.4)
Fourth	1	1	1	1
Source of information				
Electronic mass media	-	2.9(1.2–2.2)*	1.5(0.5–4.2)	4.3(1.6–11.7)*
School	-	3.2(1.0–10.3)	7.3(0.8–62.1)	9.7(3.2–28.9)*
Friends or neighbors		5.1(0.6–44.0)	1.1(0.2–6.1)	2.6(0.6–11.3)
Newspaper	1	1	1	1

*Statistically significance at 0.5 level.

## Discussion

This study shows that the majority (77.5%) of heath science students were aware of climate change. This can serve as a starting point for engaging health sciences students in addressing climate change and perhaps even broader environmental issues in relation to public health. This high awareness level was similar to those reported for adults by two studies in Europe (88.0%) and the United States (82.0%) but higher than in a study in various countries in sub-Saharan Africa, where an average of 44.0% of adults were aware of climate change impacts [24]. The differences between our and the other sub-Saharan studies may largely be due to differences in study populations. Thus high awareness can be expected among health sciences students who were more highly educated than the general population in the other Sub-Saharan study.

We found a slightly higher proportion of female students (78.6%) than male students (76.7%) to be aware of climate change and its health impacts, although these differences were statistically not significant. Similarly, a study conducted in the United States by McCright [25] found that women had a better understanding of the impact of climate change than men, and the review by Roehr [26] concluded that women generally recognize climate change as a more severe threat than men. These gendered differences may be attributed to the relatively higher social responsibility of women than men in their communities, which increases their understanding of the causes and consequences of climate change [27]. Moreover, women not only had relatively good knowledge of climate change but also the capability to change their environment [28]. This pattern of female awareness and their potential contribution to reducing climate change and mitigating impacts is contrary to the common perception that African females are unaware of environmental issues and helpless to deal with them. There is thus a need for the active participation of women in debates on mitigation and adaptation to climate change policy that needs to be recognized by policy makers.

This study also shows that the awareness of climate change varies in accordance with age and the home region of study participants. The general knowledge of climate change was higher among the older than younger students. This finding differs from a Cambodian study of the general population [29], which may be explained by the fact that our study sample was selected on the basis of education. Students from areas vulnerable to climate change were more aware of general climate change impacts than students from less impacted areas. These results also differ from those of the above Cambodian study, where the spatial differences were smaller [29]. These differences may reflect the relatively wider experiences of Ethiopian students from areas experiencing frequent heat waves, drought, or flooding.

The majority of the students identified flooding as the main climate change impact, followed by increasing temperatures and drought. Similarly, a study by Abaya et al. [19] of Ethiopian health officials in Somali Region indicated that flooding is one of the major climate change impacts. Flooding has a direct impact on human health, causing injuries and death during flooding. Similarly, increasing temperatures pose a risk for heat stroke (heat stress) [1,3,9,10]. However, flooding may be seen as a particularly serious impact of climate change in this specific study because of the loss of life and propriety damage that was recently reported form different parts of Ethiopia [14].

Electronic mass media was cited as the dominant source of information on climate change. Similarly, a study of the general population in northwestern England showed that most respondents had obtained information on climate change issues from electronic mass media [Northwest Regional Development Agency: Perceptions of climate change within the Northwest 2009 study, unpublished report].

University courses/curriculum is the best option to prepare health sciences students to effectively address health impacts of climate change because the information received through mass communication is not sufficient to prepare health sciences students to effectively design interventions to tackle health effects of climate change. Nevertheless, health sciences students need a more comprehensive curriculum on climate change and its impacts on health. The low attention given to climate change related health impacts in the College of Health Sciences of Haramaya University is insufficient to adequately prepare students to effectively plan, implement and evaluate interventions aimed at preventing and ameliorating these impacts.

This study found that while slightly more than half (52.3%) of the students stated that climate change could have an impact on human health the remaining students either did not make this association or were uncertain. This finding differs from a study in the Somali Region of Ethiopia, where 80.1% of the local health officials were aware of the role of climate change in human health [19]. These differences may be due to the fact that Somali Region is highly prone to climate change and climate variability [30], where health officials may thus be highly aware of this problem. But Abaya et al. [19] pointed out that even though the local health officials in Somali Region were aware of the health impacts of climate change most of them did not have a good understanding about this relationship. This appears to be due to lack of information on this subject provided by schools and points to the need to include information on the relationship between climate change and health in public health courses.

Heat stroke was the most frequently mentioned climate change impact (Figure 2), which is consistent with the findings of a study of the general population in Malta [20]. Very few participants in the present study knew about the indirect effects of climate change on mental health, such as anxiety and stress or on scabies and trachoma (Figure 2). Several other studies identified mental health problems, such as post-traumatic stress disorder or depression associated with extreme weather events as common disorders resulting from climate change [3,31]. The complex relationship between catastrophic weather events and mental health is often underestimated in disaster areas, where physical injuries and economic damages are given top priority, indicating the need to more fully assess the indirect and possibly more persisting impacts on mental health [31].

Two hundred and sixty-nine participants (87.9%) claimed that they lacked the necessary knowledge to address climate change related health impacts. This rate is much lower than in a study carried out in the USA, where about two-thirds of 133 local health officials claimed to have adequate knowledge to respond to climate change heath impacts [21]. This difference may be due to the better preparedness of local health officials, the availability of more resources, and closer cross-sectoral cooperation between the health and meteorology sectors in the USA [21]. In Ethiopia, where adequate resources are lacking and levels of preparedness are low [19], strengthening of the programs of health sciences schools is a necessary and urgent step towards strengthening the nations capacity to successfully deal with climate change impacts. We found that more than half (53.6%) of the (306) respondents perceived climate change as a very serious risk to human health (Figure 2). These results are similar to those of a study in California, where 56.0% of local health officials perceived climate change impacts to constitute a serious risk to human health [32].

Medical students (70.4%) and pharmacy students (60.7%) were slightly less knowledgeable of climate change impacts than health officer students (84.8%). These differences might be due to the fact that the nature of medical and pharmacy programmes were designed to prepare the students primarily for clinical medicine rather than public and environmental health.

The study shows a strong positive association (P < 0.05) between students’ awareness of climate change and their perceived departmental concern for preventing the public health consequences of climate change related health impacts. This indicates that students who are better informed about climate change impacts in their department are more likely to address them. These results are consistent with findings of studies in the United Kingdom and Malta of the general population (Northwest Regional Development Agency: Perceptions of climate change within the Northwest 2009 study, unpublished report, [20]).

Our finding that students who knew about climate change were more likely to be aware about health impacts of climate changes suggests that providing training about this relationship may encourage heath science students to learn more about the health related risks of climate change. This again points out the need to increase students’ knowledge on climate change and health linkages through climatology courses covering climate change to facilitate their involvement in preventive programs and actions addressing this problem (Table 4).

The present study also reveals that students who reported obtaining information from friends or neighbors were more likely to know about climate change impacts than those study participants who relied on newspapers. This suggests that students may learn more from discussions with peers, social networks, proximal relations, and social gatherings than from newspapers, which tend to neglect cultural and social issues in their reporting, although low access to newspapers also impedes their use in Ethiopia. Further studies of the role of peer groups and other social networks may facilitate the development of strategies to increase students’ knowledge of climate change impacts.

### Limitation of the study

This study did not review the curriculum of Haramaya University’s health sciences departments; precluding a full assessment of course content related to climate change issues. This information is needed for the full evaluation of student knowledge and the search for curriculum and instruction-based solutions to knowledge deficiencies. It is an enormous challenge in trying to compare this study within identical studies. This could also limit the generalizability of this study for comparative purposes at the national and international levels. Nevertheless, the widespread occurrence of climate change in Sub-Saharan Africa suggests that the approach used may be appropriate and encourage the development of curricula appropriate for the study of health impacts of climate change at other universities in the region.

## Conclusion

The results of this study reveal that significant numbers of health science students at Haramaya University were aware of climate change and its health impacts but that most of them reportedly lacked the necessary knowledge to address the health impacts of climate change. The study also shows that many more students had gained information on climate change related health impacts from electronic mass media than from the university. This indicates the need for relevant courses on the climate change/human health interface be included in the curriculum of Haramaya University. Moreover, the strong positive association between students’ awareness of climate change and their departmental affiliation should be harnessed to educate students on the prevention of climate related public health impacts. The results of this exploratory study of climate related health impacts in Ethiopia provides baseline data and may serve as a model for future studies at Haramaya and other universities. Although most findings of this study corroborate those of general populations, further studies at other Ethiopian universities are required to validate our findings in student populations and inform planners and administrators responsible for health science curriculum development.

Although this study did not review the curriculum of Haramaya University’s Health Science College, the authors conclude that curriculum changes may have to be considered to increase the knowledge of students of the climate change/health nexus. Follow-up studies may reveal whether curricular changes are required to achieve satisfactory knowledge of health science students at graduation. Additional studies are also urgently needed in schools of health sciences at other Ethiopian universities to generate a wider data base at the national level on students’ understanding of the relevance and dynamics of the climate change/health relationship. Our findings also call for greater awareness among Ethiopian and other African health science professionals of climate change-linked health impacts because of the continent-wide occurrence of this increasingly recognized and documented environmental health problem.

## Competing interests

The authors declare that they have no competing interests.

## Authors’ contributions

NA developed the idea of this study, formulated the study design, gathered the data, carried out most of the statistical analysis and assisted with preparing the manuscript. BA was involved in developing the manuscript and assisted with the statistical analysis. HK edited the manuscript. All authors read and approved the final manuscript.

## Pre-publication history

The pre-publication history for this paper can be accessed here:

http://www.biomedcentral.com/1471-2458/14/587/prepub
